# No Evidence of Novel Respiratory Viruses on Two Texas Dairy Farms Before the H5N1 Avian Influenza Virus Epizootic

**DOI:** 10.1111/irv.70146

**Published:** 2025-07-30

**Authors:** Laura A. Pulscher, Lyudmyla V. Maruschak, Ismaila Shittu, Hisham Alsharif, Gregory C. Gray

**Affiliations:** ^1^ Department of Medicine (Division of Infectious Diseases) University of Texas Medical Branch Galveston Texas USA; ^2^ Department of Microbiology and Immunology University of Texas Medical Branch Galveston Texas USA; ^3^ Institute for Human Infections and Immunity University of Texas Medical Branch Galveston Texas USA

**Keywords:** coronaviruses, dairy cattle, influenza A virus, influenza D virus, zoonoses

To the editor:

Livestock intensification and modern farming practices, such as confinement and increased livestock densities, are thought to be strongly linked to zoonotic disease emergence and amplification [1, 2]. This may favor increased transmission, particularly to those working in close contact with livestock [1]. The recent introduction and spread of H5N1 avian influenza virus into dairy farms and dairy farm workers in early 2024 [3] highlights a need for surveillance of emerging zoonotic respiratory diseases at the cattle worker–cattle interface. In addition to influenza A viruses (IAV), influenza D virus [4] (IDV) and bovine coronaviruses [5] (BCoVs) are reservoired in cattle and may spill over into other animals, including sometimes to humans. For example, recent molecular and serological evidence suggests IDV may be spilling over into humans, specifically those with close contact to cattle [6, 7, 8]. Similarly, BCoVs have a large host range, and most notably, the seasonal human coronavirus, OC43‐CoV, is thought to have originated from cattle, sharing a 96% global nucleotide identity with BCoV [9]. To this end, this one health‐oriented study sought to determine IAV, IDV, and coronavirus transmission on dairy farms. We did so by prospectively collecting samples from cattle workers, their cattle, and the dairy farm environment to better understand the epidemiology and ecology of IAV, IDV, and coronaviruses.

From December 2022 to December 2023, we prospectively collected samples from 53 dairy workers, 60 dairy cows, 30 bioaerosol samples, and 22 dust samples from farm environments on two dairy operations in Texas. With farm owners' approval, dairy farms were visited every 3–4 months over the course of the study. After obtaining signed consent, nasal washes were collected from cattle workers by injecting 5 mL of sterile water into one nostril and collecting the expressed fluid. Participants also permitted the collection of up to 10 mL of whole blood. Within 12 h of collection, blood was spun down, and sera aliquoted and placed on ice. Nasal swabs were also collected from five cows at each encounter and up to three cows each month in‐between encounters, prioritizing cows with signs of respiratory illness. Briefly, animal technicians on farms placed 6‐in. polyester swabs into the nare of a cow and then placed the swab into 3 mL of viral transport media (Huachenyang [Shenzhen] Technology Co. Ltd. or Rocky Mountain Biologicals LLC) samples were placed on ice or shipped on cold packs to UTMB for analysis.

Environmental sampling, including bioaerosol and dust sampling, was also conducted at each farm encounter. National Institute for Occupational Safety and Health (NIOSH) bc251 multi‐stage bioaerosol samplers were placed in four locations on each farm where humans and cows were in close contact or where sick cows were located. Samplers were placed in a central location at breathing height, where possible, and run for 3–4 h at a flow rate of 3.5 L/min and processed as previously described [10]. A liquid cyclonic bioaerosol collector (Midwest Micro‐Tek, Brookings, SD, USA) capable of a flow rate of 400 L/min was also placed in milking parlors. Ten milliliters of minimum essential medium (MEM; Gibco, Billings, MT) was placed into collectors, run for 30 min, then MEM was removed and immediately placed on ice. Dust samples were collected by wiping approximately 1 ft^2^ areas of different hard surfaces (walls, railings, pipes) located within 1 m of NIOSH air samplers with 8 × 8 in Nalgene Super Versi‐Dry Surface Protector wipes (Thermo Scientific Nalgene). Twenty milliliters of PBS was added to each bag with dust wipes, hand mixed for 2–3 min, and then PBS was squeezed off dust wipes and kept on ice. All samples were kept on ice and transported to the UTMB One Health Laboratory within 72 h for processing and stored at −20°C (sera) or −80°C (all other samples) until further analysis. Ethical oversight for human and animal sampling was provided by UTMB (IRB Protocol #22‐0181 and IACUC Protocol # TEMP‐0523).

Viral RNA extraction was conducted using a QIAamp Viral RNA Mini Kit by hand or on a QIACube Connect (Qiagen, Valencia, CA, USA) per the manufacturer's instructions. Samples were then analyzed with a real‐time RT‐PCR (qRT‐PCR) screening assay targeting the Matrix gene for IAV [11] (human and cattle samples) and the NP [12] and PB1 [13] genes for IDV (all samples) using AgPath‐ID One‐Step RT‐PCR Reagents (Applied Biosystems, Waltman, MA). A gel‐based conventional semi‐nested RT‐PCR targeting the RNA‐dependent RNA polymerase (RdRp) genome was also performed for coronaviruses [14] using Superscript III Platinum One‐Step RT‐PCR System with Platinum Taq DNA Polymerase (Thermo Fisher Scientific Inc., Waltham, MA) and Platinum Taq DNA Polymerase (Invitrogen). Amplicons were sent for sequencing and assessed for sequence similarity to other viruses using the National Center for Biotechnology–Basic Local Alignment Search Tool (NCBI BLAST).

Using a recombinant H5N1 virus (Rg‐A/bald eagle/Florida/W22‐134‐OP/2022 PR8‐H5N1), a microneutralization assay (MN) was performed on receptor‐destroying enzyme II (Denka Seiken, Tokyo, Japan) pre‐treated human serum samples following standard protocols [15]. Similarly, MN for IDV antibodies was performed using influenza D Kansas strain (D/Bovine/Kansas/1‐35/2010) as previously described [7].

Of the 89 samples tested, three cattle workers (3.4%) had molecular evidence of coronaviruses in nasal washes. NCBI BLAST analyses of these three sequences showed a close identity to SARS‐CoV‐2/human/VNM/T1HN/2022 (NCBI accession number ON365836.1) (Table 1). No other samples had evidence of IAV, IDV, or CoVs (Table 1). Molecular evidence of SARS‐CoV‐2 was identified in three cattle workers during one farm visit, which may have indicated a small outbreak of SARS‐CoV‐2 on the farm at that time. Despite us studying dairy farms over the course of 1 year, we did not detect IAV, IDV, and other CoVs in collected samples. Considering the spillover of H5N1 avian influenza virus into dairy cattle likely occurred in March of 2024 [3], it is not too surprising that we did not detect IAV in humans or dairy cattle sampled from December 2022 to December 2023. Despite previous studies reporting a prevalence range of 2.4%–18% of IDV [16] and an incidence rate of 15.0%–70.0% for BCoVs [5] in cattle populations in the United States, we did not detect IDV or BCoVs among our sampled dairy cattle. It is possible that these viruses are not circulating in these populations, the prevalence of these viruses is low among these farms, or that infection was missed among cattle, as most of the cattle sampled were adults and the highest rates of infection for IDV and BCoVs tend to occur in calves less than 1 year old [5, 16]. Similarly, there was no evidence of IDV or novel CoVs among cattle workers sampled in this study, which could be due to the absence, or a low prevalence, of these pathogens among cattle sampled at the same time. Future studies should focus on the cattle‐cattle worker interface to fully understand the threat of emerging zoonotic viruses spilling over into humans.

**TABLE 1:** Summary of total samples tested for molecular and serological evidence of influenza A virus (IAV), influenza D virus (IDV), and coronaviruses (CoV) among cattle (*n* = 60), cattle workers (*n* = 53), and the cattle farm environment in Texas cattle farms.

Sample type	Total samples tested	IAV (+)	IDV (+)	CoV (+)
Cattle nasal swabs	60	0 (0.0%)	0 (0.0%)	0 (0.0%)
Human nasal washes	89	0 (0.0%)	0 (0.0%)	3 (3.4%)
Human sera^a^	68	0 (0.0%)	0 (0.0%)	—
Bioaerosols	30	—	0 (0.0%)	0 (0.0%)
Dust samples	22	—	0 (0.0%)	0 (0.0%)

^a^ Human sera were tested with microneutralization assays against H5N1 and influenza D virus.

## Author Contributions

**Laura A. Pulscher:** conceptualization, investigation, writing – original draft, methodology, validation, visualization, writing – review and editing, formal analysis, project administration. **Lyudmyla V. Maruschak:** investigation, validation, visualization, writing – review and editing, methodology. **Ismaila Shittu:** methodology, validation, investigation, writing – review and editing. **Hisham Alsharif:** methodology, investigation, writing – review and editing. **Gregory C. Gray:** conceptualization, methodology, investigation, formal analysis, supervision, funding acquisition, visualization, project administration, resources, writing – review and editing.

## Conflicts of Interest

The authors declare that they have no conflict of interest.

## Peer Review

The peer review history for this article is available at https://www.webofscience.com/api/gateway/wos/peer‐review/10.1111/irv.70146.

## Data Availability

The data that support the findings of this study are available from the corresponding author upon reasonable request.
